# Efficacy of Step-by-Step (SbS) in Improving Depressive Symptoms and Functional Impairment: A Systematic Review and Meta-Analysis of Randomized Controlled Trials

**DOI:** 10.1192/j.eurpsy.2025.1420

**Published:** 2025-08-26

**Authors:** V. Astori, F. Pesente, G. de Oliveira e Souza, T. Y. Kimura, G. Coachman Hollenstein, L. Pontes de Oliveira, D. Fernandes Holanda

**Affiliations:** 1 Escola Superior de Ciências da Santa Casa de Misericórdia de Vitória; 2 Universidade Federal do Espírito Santo, Vitória; 3 Universidade Federal de Minas Gerais, Belo Horizonte; 4 Universidade Federal do Amazonas, Manaus, Brazil

## Abstract

**Introduction:**

The growing need for effective solutions to bridge the mental health treatment gap is particularly critical in countries where economic and political crises have exacerbated existing mental health challenges. In this context, the urgency for scalable and accessible interventions is evident. Step-by-Step (SbS), a guided digital self-help program developed by the World Health Organization (WHO), has been implemented as a promising approach to alleviate depressive symptoms and improve functionality in vulnerable populations.

**Objectives:**

This meta-analysis aims to evaluate the findings of studies that examined the efficacy of SbS, compared with enhanced care as usual (ECAU), in reducing depressive symptoms and functional impairment.

**Methods:**

We systematically searched PubMed, Cochrane, and Scopus for randomized controlled trials (RCTs) comparing SbS with ECAU. The pooled outcomes were the overall improvement in depressive symptoms, measured by the Patient Health Questionnaire (PHQ-9), as well as functional impairment measured by the WHO Disability Assessment Schedule-12 (WHODAS). We calculated the mean difference (MD) for the outcomes, with 95% confidence intervals (CIs). Statistical analysis was performed using Review Manager (RevMan) 8.1.1 with a fixed-effect model. Heterogeneity was assessed using the I² statistic.

**Results:**

Three RCTs were included, encompassing 604 patients, of whom 263 (43.5%) participated in SbS. The population consisted of 35.1% males and 64.9% females. The mean age was 28.8 years, with a standard deviation of 8.7. SbS reduced PHQ-9 scores (MD = -3.48; 95% CI [-4.44, -2.52]; P < 0.00001; I² = 3%; Figure 1) and WHODAS scores (MD = -3.37; 95% CI [-4.84, -1.90]; P < 0.00001; I² = 0%; Figure 2) compared with ECAU.

**Image 1:**

**Figure:**
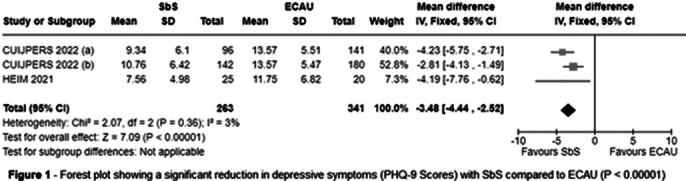

**Image 2:**

**Figure:**
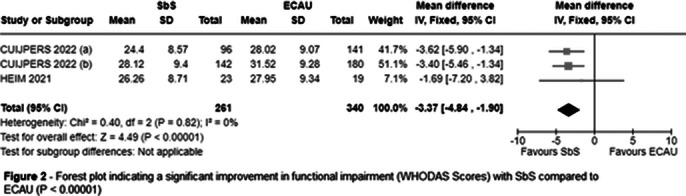

**Conclusions:**

This meta-analysis of RCTs suggests that SbS has a positive effect in reducing depressive symptoms and functional impairment compared with ECAU.

**Disclosure of Interest:**

None Declared

